# Advances in Imaging and Diagnosis of Abdominal Aortic Aneurysm: A Shift in Clinical Practice

**DOI:** 10.7759/cureus.81321

**Published:** 2025-03-27

**Authors:** Abubakar I Sidik, Malik K Al-Ariki, Abdulmajid Ilyas Shafii, Md Limon Hossain, Farjana Najneen, Gulten Ak, Derrar Ahlam, Abdoli Shakiba, Debraj Ghosh, MST Asia Ajgar Bithi, Mussalim I Kairatuly

**Affiliations:** 1 Cardiovascular Surgery, Peoples' Friendship University of Russia, Moscow, RUS; 2 Cardiothoracic Surgery, Peoples' Friendship University of Russia, Moscow, RUS; 3 Cardiovascular Medicine, Peoples' Friendship University of Russia, Moscow, RUS; 4 Cardiology, First Moscow State Medical University, Moscow, RUS

**Keywords:** abdominal aortic aneurysm, artificial intelligence, computed tomography angiography, imaging, risk stratification, screening programs, ultrasonography

## Abstract

Abdominal aortic aneurysm (AAA) is a potentially life-threatening vascular condition, with rupture carrying a high mortality risk. Advances in imaging technologies have significantly improved the detection, risk stratification, and management of AAA, necessitating periodic updates to international clinical guidelines. This review examines recent advancements in AAA imaging modalities, their role in diagnosis and risk assessment, and evolving screening strategies in response to changing epidemiological trends. A systematic literature search was conducted following Preferred Reporting Items for Systematic Reviews and Meta-Analyses (PRISMA) guidelines, identifying key studies on diagnostic imaging techniques such as ultrasonography (US), computed tomography angiography (CTA), magnetic resonance angiography (MRA), intravascular US (IVUS), positron emission tomography-computed tomography (PET-CT), and artificial intelligence (AI)-driven imaging.

US remains the preferred screening tool due to its cost-effectiveness, while CTA is the gold standard for preoperative planning. MRA is an alternative for patients with renal impairment, and emerging technologies such as AI-based imaging, IVUS, and PET-CT enhance risk prediction and surgical decision-making. Declining AAA prevalence, attributed to reduced smoking rates and improved cardiovascular risk management, has led to a shift from population-wide screening to targeted screening based on individual risk factors. Additionally, the standardization of imaging protocols and caliper placement techniques is crucial for accurate measurements and consistent clinical decision-making. As imaging technology continues to evolve, integrating AI, genetic markers, and biomarkers into screening and diagnostic protocols may enable more precise and personalized AAA management. Future research should focus on refining imaging-based risk stratification models to optimize screening and treatment strategies.

## Introduction and background

An abdominal aortic aneurysm (AAA) is a localized dilation of the abdominal aorta, typically defined as an aortic diameter exceeding 3.0 cm or increasing by more than 50% compared to the normal size (1.4 to 3.0 cm) [1,2]. It is a potentially life-threatening condition, as the progressive weakening of the arterial wall can lead to rupture, which is associated with high mortality. The primary risk factors for AAA development include age, male sex, smoking history, hypertension, and a family history of the disease [3,4]. While many AAAs remain asymptomatic until rupture, early detection through imaging plays a crucial role in preventing adverse outcomes.

Timely diagnosis and risk stratification are essential for managing AAA and preventing rupture-related mortality, which remains high despite advances in surgical techniques. Small AAAs, measuring between 3.0 and 5.4 cm, are generally monitored through regular surveillance. In comparison, larger aneurysms (≥5.5 cm in men and ≥5.0 cm in women) often require elective repair due to the increased risk of rupture [5]. The European Society for Vascular Surgery (ESVS) emphasizes the importance of balancing the risks of surgery against the risk of aneurysm rupture by implementing evidence-based screening and surveillance strategies tailored to individual patient profiles [6].

Over the past few decades, imaging techniques have significantly evolved, improving AAA's detection, risk assessment, and management. Historically, AAAs were diagnosed through abdominal palpation or incidentally during autopsy, which provided a rare opportunity to detect AAAs post-mortem, particularly in individuals who died suddenly or from non-specific abdominal or cardiovascular symptoms. This led to a high number of undetected cases [7,8]. The introduction of ultrasonography (US) in the 1970s revolutionized AAA screening, providing a cost-effective, non-invasive, and highly sensitive diagnostic tool [9]. However, its limitations led to the development of more advanced imaging modalities, such as computed tomography angiography (CTA), which became the gold standard for detailed vascular assessment in the 1980s, offering high-resolution images for preoperative planning [10]. Although CTA provides excellent anatomical detail, concerns about radiation exposure and contrast-induced nephropathy led to the emergence of magnetic resonance angiography (MRA) in the 1990s as a viable option for patients with renal impairment. Still, its high cost and limited accessibility have restricted widespread use [11]. Recent advances in AI-enhanced imaging, intravascular US (IVUS), and positron emission tomography-computed tomography (PET-CT) have further refined AAA detection and risk prediction, paving the way for more precise, personalized management strategies [12,13].

The increasing complexity of AAA diagnosis and treatment underscores the necessity of regularly updating international clinical practice guidelines (ESVS, Society for Vascular Surgery (SVS), and American College of Cardiology/American Heart Association (ACC/AHA)) to align with technological advancements and emerging evidence. All the major guidelines continuously undergo yearly changes to meet recommendations based on the results of new studies.

This article reviews advancements in AAA imaging and diagnosis, emphasizing updates in international guidelines. It evaluates imaging techniques such as US, CTA, MRA, AI-driven imaging, IVUS, and PET-CT, analyzing their benefits for risk stratification and patient management. Emerging technologies like AI-based models, 3D reconstruction, and hybrid imaging are explored for their role in personalized screening. The article also highlights tailored screening protocols based on patient risk factors. It identifies research gaps, including AI standardization, novel contrast agents, and wearable US devices, aiming to bridge current knowledge with future trends in AAA imaging and diagnosis.

## Review

Methodology

A systematic electronic literature search was conducted on February 22, 2025, using multiple databases, including PubMed, Web of Science, Scopus, and the Cochrane Library. The search was performed with the following query: "abdominal aortic aneurysm" AND ("imaging" OR "diagnosis") AND ("ultrasonography" OR "computed tomography angiography" OR "CTA" OR "magnetic resonance angiography" OR "MRA" OR "intravascular ultrasound" OR "IVUS" OR "positron emission tomography-computed tomography" OR "PET-CT").

To ensure compatibility with each database, the search string was modified accordingly. The literature search followed the Preferred Reporting Items for Systematic Reviews and Meta-Analyses (PRISMA) 2020 guidelines, incorporating the checklist and flow diagram [14]. Studies were included if they met the following criteria: they focused on diagnostic imaging for AAA, were case reports, original research, meta-analyses, review articles, or clinical guidelines, were published in English, and had been published since 2020; this time range was chosen to analyze the most recent scientific work on the subject matter. Any studies that did not meet these predefined criteria were excluded from the review.

The selection process was conducted by two independent reviewers, each of whom assessed the titles and abstracts of the retrieved articles to determine whether they met the inclusion criteria. To evaluate study quality and potential bias, the authors used the Critical Appraisal Skills Programme (CASP) checklist for qualitative research, which consists of 10 key assessment questions [15]. The inclusion of each article in the study was based on the consensus reached by both reviewers.

The identification phase begins with 552 records retrieved from multiple databases such as PubMed, Web of Science, Scopus, and the Cochrane Library. After removing duplicate records, 478 unique articles remain for screening. Removing duplicates is crucial to avoid overrepresentation of studies.

In the screening phase, the remaining 478 records are reviewed based on their titles and abstracts to determine their relevance to the study. During this process, 192 irrelevant records that do not meet the study’s inclusion criteria are excluded. Next, in the eligibility phase, the full text of 286 articles is assessed to ensure they provide relevant information. However, 88 articles are excluded due to the unavailability of the full text, and another 88 articles are removed because they do not specifically focus on diagnostic imaging for AAA, which is the central theme of the review.

Finally, 198 studies are included in the qualitative synthesis in the inclusion phase, meaning they contribute to the narrative review by providing insights into AAA imaging techniques. A subset of 110 studies is selected for quantitative synthesis, where data is analyzed statistically to conclude.

**Figure 1:**
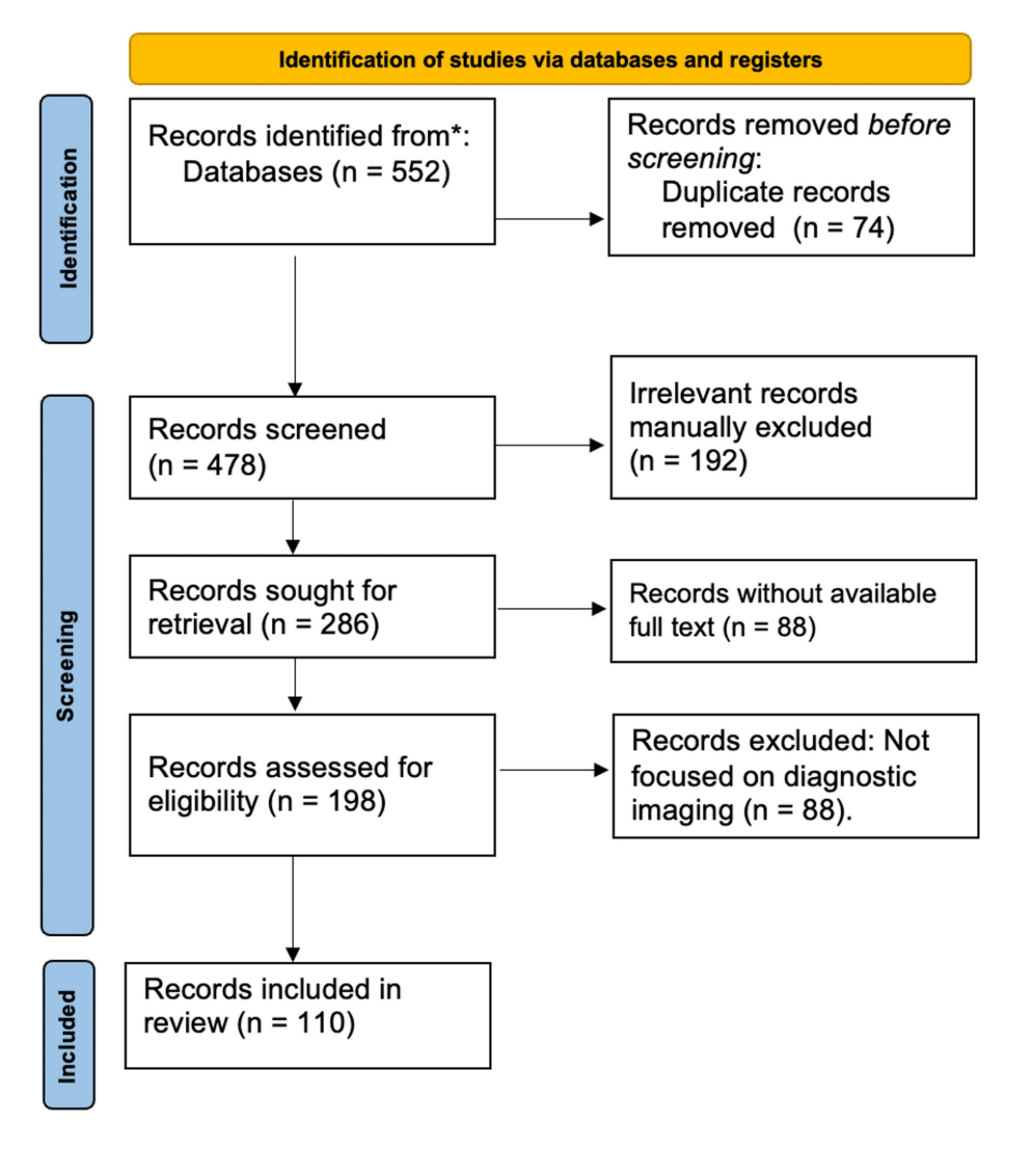
PRISMA flow diagram detailing steps in the identification and screening of sources PRISMA: Preferred Reporting Items for Systematic Reviews and Meta-Analysis

This flow diagram provides a clear and systematic overview of the study selection process, ensuring the review is conducted rigorously and transparently. By following PRISMA guidelines, the study ensures that only relevant and high-quality studies are included in the final analysis (Figure 1) [14].

Data analysis

Recommendations for Diagnosing AAA

The ESVS and SVS Guidelines emphasize a structured approach to diagnosing AAA, refining previous recommendations based on contemporary evidence. The primary diagnostic threshold remains an aortic diameter of ≥30 mm, although variations exist for different populations; lower thresholds (such as ≥27-28 mm) have been suggested for women and Asians due to their lower body mass index [6,16]. US remains the first-line imaging modality for AAA screening and diagnosis due to its non-invasive nature, cost-effectiveness, and reliability in detecting aneurysm size [9].

For confirmatory diagnosis and preoperative planning, CTA is recommended once the threshold for elective repair is met, as it provides high-resolution images of vascular anatomy [10]. MRA is considered an alternative for patients with renal impairment, but its use is limited due to availability and cost [11]. International guidelines also emphasize standardized caliper placement in imaging assessments to ensure accurate measurement and follow-up comparisons. US measurements taken during diastole can show a diameter up to 2 mm smaller than systolic measurements. Implementing a standardized US protocol incorporating ECG gating and precise caliper placement during offline analysis helps minimize measurement variability [6,17].

Current recommendations suggest that all incidentally detected AAAs should be referred to a vascular surgeon, except in cases where the patient has a very limited life expectancy. This ensures that appropriate surveillance or intervention is provided when necessary​ [6,16].

Role of Risk Factors, Screening Programs, and Incidental Detection

AAA development is closely linked to modifiable and non-modifiable risk factors. The dominant risk factors include male sex, age (>65 years), and smoking, with smoking contributing to up to 75% of AAA cases [18]. Additional risk factors include hypertension, dyslipidemia, and a family history of AAA [3]. AAA screening should be risk-stratified, focusing on high-risk populations to improve detection rates and cost-effectiveness​ [6].

Screening programs have been widely implemented in various countries, including the UK, Sweden, and the USA, demonstrating a reduction in AAA-specific mortality. In randomized trials, screening was associated with a 40% reduction in all AAA-related deaths, reinforcing its clinical value. However, the guidelines highlight the changing epidemiology of AAA, where the overall prevalence has declined due to improved cardiovascular risk management and reduced smoking rates [6].

Despite these declines, screening remains cost-effective for men aged ≥65 years, particularly current or former smokers. However, there is limited evidence supporting routine screening for women, as studies have shown a fourfold lower prevalence compared to men. The guidelines recommend that screening in women be considered only in those with a first-degree relative with AAA, given the genetic component of the disease​ [6].

AAA is also frequently detected incidentally through imaging performed for other conditions, such as CT scans for back pain or abdominal discomfort. Studies indicate that many incidentally discovered AAAs are not referred for further evaluation, underscoring the need for improved awareness among general practitioners and radiologists; automated radiology alerts or implementation of standardized reporting protocols are steps in the right direction to resolve this issue. All incidentally detected AAAs should trigger vascular surgery referral to ensure appropriate risk assessment and management​ [19,20].

Epidemiological Data Influencing Diagnosis Protocols

The global prevalence of AAA has declined over the past two decades, largely attributed to decreased smoking rates and advances in cardiovascular risk management. Data indicate that AAA is negligible before the age of 55-60 years, but its incidence increases steadily with age [21]. In 1990, the prevalence among individuals aged 75-79 years was 2,423 per 100,000, compared to 2,275 per 100,000 in 2010, with the highest rates observed in Australasia, North America, and Western Europe; this can be associated with the high life expectancy in these countries​ [21].

Screening studies have provided insight into contemporary AAA prevalence, with programs in Sweden and the UK reporting an AAA prevalence of <1% in men aged 65 years [22]. In contrast, a US-based screening program targeting only smokers reported a prevalence exceeding 5% [23]. Over the past 20-50 years, the incidence of ruptured AAA admissions and repairs has declined in many European countries and the USA, despite an aging population; this has been linked to reduced smoking rates and advancements in cardiovascular risk management, including improved blood pressure control and the widespread use of statins and antiplatelet medications [24-26].

These epidemiological trends have led to revised screening protocols, where the emphasis is shifting toward targeted screening strategies based on individual risk profiles. As a result, the present guidelines suggest that high-risk groups, including first-degree relatives of AAA patients and individuals with peripheral aneurysms, should be prioritized for screening [6,16]. The guidelines also stress the importance of continuously evaluating screening programs to ensure they remain clinically and economically viable.

Imaging Modalities in AAA Detection and Surveillance

Four randomized trials on population-based screening for AAA in men conducted in the UK, Denmark, and Australia, along with a smaller trial in women (9342 women aged 65-80) in the UK (Table 1) [6], demonstrated a significant reduction in AAA-specific mortality [27-30]. At the longest follow-up reported in each trial, the group invited for screening showed a statistically significant decrease in all-cause mortality, with a risk ratio of 0.987 (p=0.03).

**Table 1:** Summary of randomized trials on population-based screening for AAA in men Note: The participation rate in the Western Australia study is based on the percentage of individuals who were alive when they received an invitation for screening. In some cases, randomization preceded the invitation by several months. MASS: multicenter aneurysm screening study, OR: odds ratio, CI: confidence interval, AAA: abdominal aortic aneurysm, UK: United Kingdom Thompson et al. [28]; Scott et al. [29]; Ashton et al. [30]; Lindholt et al. [31]; Norman et al. [32]

Trial characteristics	Chichester, UK [29]	Viborg, Denmark [31]	MASS, UK [28,30]	Western Australia [32]
Participants enrolled	15,775	12,639	67,770/67800	41,000
Population	Men and women	Men	Men	Men
Age range (years)	65–80	65–73	65–74	65–79
Recruitment period	1988–1990	1994–1998	1997–1999	1996–1998
Publication year	1995	2005	2002	2004
Screening participation (%)	68	76	80	70*
AAA detection rate (%)	4 (7.6% in men)	4	4.9	7.2
Screening location	Hospital	Hospital	Community	Community
Intervention criteria	Aneurysm ≥60 mm	Aneurysm ≥50 mm (external diameter)	Aneurysm ≥55 mm (internal diameter)	No specific threshold
Average follow-up (years)	4.1	13.0	13.1	12.8
AAA-related mortality (OR, 95% CI) for screened vs. non-screened	0.59 (0.27–1.29) (men only)	0.31 (0.13–0.79)	0.58 (0.42–0.78)	0.91 (0.68–1.21)
All-cause mortality (OR, 95% CI) for screened vs. non-screened	1.07 (0.93–1.22) (men only)	0.98 (0.95–1.02)	0.97 (0.93–1.02)	0.98 (0.96–1.01)

US as the primary screening tool: US remains the first-line imaging modality for the screening and surveillance of small AAAs. It is particularly recommended for high-risk groups, including men over 65 years and individuals with a family history of AAA. US is the most effective, non-invasive, and cost-efficient screening tool for AAA detection​ [9].

**Figure 2:**
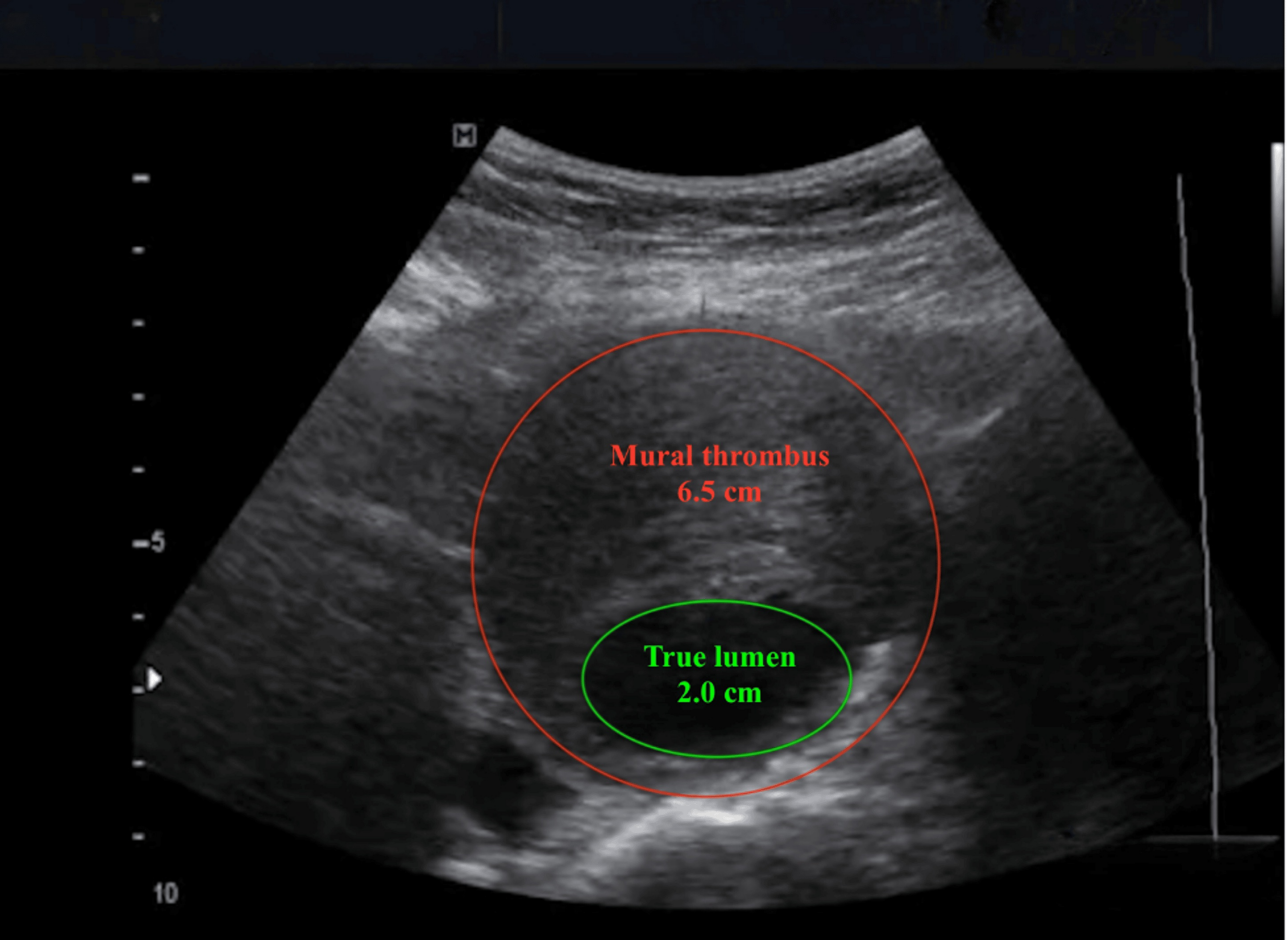
AAA with a narrowed lumen due to large thrombotic mass partially occluding it AAA: abdominal aortic aneurysm Authors' original image

For surveillance, US is recommended for patients with small AAAs measuring 30-54 mm in diameter, with monitoring intervals determined by aneurysm size. The standard recommendation suggests that AAAs measuring 30-39 mm should be monitored every three years, while those between 40-49 mm require annual surveillance. For AAAs measuring 50-54 mm, US should be performed every six months, as these aneurysms are approaching the threshold for surgical intervention (Figure 2) [5].

Accurate diameter measurement in US is crucial, and anteroposterior plane measurement with consistent caliper placement is preferred for standardization and reproducibility. However, US has limitations, including operator dependency, difficulty in obese patients, and reduced accuracy in cases of severe arterial calcification; harmonic imaging or contrast-enhanced US would be very beneficial in these instances​ [17].

CTA: CTA is the gold standard for AAA diagnosis and preoperative planning, providing high-resolution imaging of the aorta and its branches. It is particularly useful when aneurysm size approaches the surgical threshold or when planning endovascular repair. CTA is for confirming AAA diagnosis, preoperative evaluation, and rupture assessment​ [10].

Advancements in CTA include using contrast-enhanced imaging techniques, which improve visualization of aneurysm morphology and help identify intraluminal thrombus and arterial wall integrity [31]. 3D reconstruction and AI-assisted imaging analysis (e.g., U-Net convolutional neural network model) are emerging tools that enhance risk prediction models and aid surgical planning [32]. These developments allow for more precise diameter measurements, volumetric analysis, and dynamic flow assessments, leading to better treatment strategies. One of the primary concerns with CTA is radiation exposure and contrast-induced nephropathy, particularly in patients with chronic kidney disease. Strategies to minimize radiation exposure include low-dose protocols (e.g., tube voltage, dual-energy CT scan, contrast dilution) and alternative contrast agents (e.g., iodixanol, CO2), which are being increasingly adopted in clinical practice [33,34].

MRA: MRA is an alternative imaging modality that provides high-resolution vascular imaging without radiation exposure. It is particularly useful for patients with renal impairment, as it reduces the risk of contrast-induced nephropathy associated with CTA. International guidelines recommend MRA as a second-line option when CTA is contraindicated​ [6,11,16].

A key advantage of MRA is its ability to differentiate between stable and high-risk aneurysms using functional MRI techniques. These methods (though yet to be validated) assess aortic wall stress, inflammation, and biomechanical properties, potentially improving rupture risk prediction [35]. However, MRA remains less accessible and more expensive than CTA, and its role in routine AAA diagnosis remains limited to specific cases​ [11].

Emerging imaging techniques: New imaging technologies enhance AAA detection and risk stratification. IVUS is increasingly used to assess aortic wall integrity and aneurysm morphology, particularly in endovascular aneurysm repair (EVAR) procedures [12,36,37]. It provides real-time imaging and allows for precise endograft placement, reducing the risk of endoleaks.

PET-CT and molecular imaging have shown promise in detecting inflammatory aneurysms and identifying high-risk cases. PET-CT imaging can assess metabolic activity in the aortic wall, helping distinguish between stable and rupture-prone aneurysms. For example, when a continuous increase in aneurysm size is observed post-EVAR with no associated endoleak, PET-CT can be applied to rule out occult infection or the presence of a vascular malignancy [12,13]. These imaging techniques are still in the early stages of clinical adoption but may play a significant role in future AAA management.

Artificial intelligence (AI) and machine learning applications are also being integrated into AAA imaging, particularly for automated detection, segmentation, and risk prediction. AI-driven algorithms can analyze large imaging datasets, improving diagnostic accuracy and predicting aneurysm growth and rupture risk more effectively than traditional methods​ [38].

Integrating AI standardization, advanced contrast agents, and wearable US represents a significant leap forward in AAA imaging and risk assessment. Standardized AI models will enhance diagnostic consistency, novel contrast agents will provide deeper physiological insights, and wearable US will enable continuous, patient-specific monitoring. Together, these innovations will bridge current knowledge gaps and pave the way for a future of precision medicine in AAA management [33,34,38].

Controversies in AAA Measurement: The Caliper Placement Debate

Accurate measurement of AAA diameter is crucial for monitoring aneurysm progression and determining the optimal timing for surgical intervention. However, inconsistencies in caliper placement techniques have led to significant variations in reported aneurysm sizes, contributing to uncertainty in clinical decision-making. The SVS and ESVS guidelines emphasize the need for standardized measurement protocols to minimize discrepancies and ensure consistency across different imaging modalities and institutions [6,17,19].

**Figure 3:**
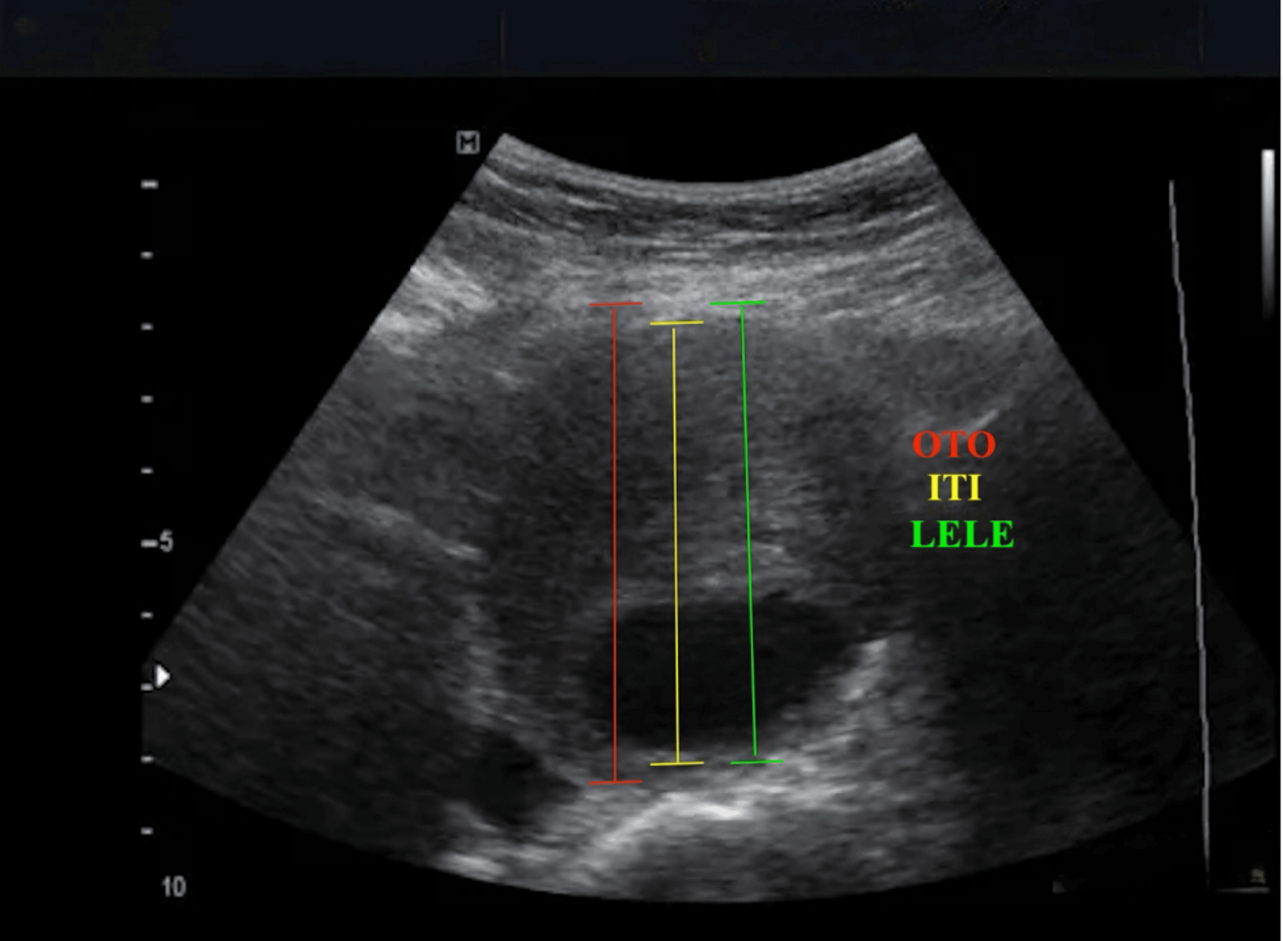
Caliper placement for measurement of aortic diameter OTO: outer to outer, ITI: inner to inner, LELE: leading edge to leading edge Authors' original image

Variability in AAA diameter measurement: The measurement of AAA diameter can vary depending on the caliper placement technique, with the most common methods being outer-to-outer diameter, inner-to-inner diameter, and leading edge-to-leading edge measurement, as shown in Figure 3 [17]. The outer-to-outer technique measures the aneurysm by placing calipers on the external walls of the aorta. In contrast, the inner-to-inner technique focuses on the luminal borders, excluding the aortic wall thickness and thrombus. These variations can lead to differences in reported aneurysm size, directly impacting clinical decision-making, particularly regarding surgical intervention thresholds [39,40].

The choice of measurement technique also influences the longitudinal tracking of aneurysm growth. If different caliper placement methods are used over time, it may create a false impression of rapid expansion or stability, leading to inappropriate clinical decisions [39,40]. Consistency in measurement methodology is paramount, so each institution should adopt a standardized approach to ensure reliable tracking of aneurysm progression.

Clinical implications of measurement inconsistencies: Measurement discrepancies in AAA diameter can have significant clinical implications, particularly in the decision to proceed with elective repair. Overestimation of aneurysm size may lead to premature surgical intervention, exposing patients to unnecessary perioperative risks, while underestimation can result in delayed repair, increasing the risk of rupture [39]. The threshold for elective intervention, typically ≥5.5 cm for men and ≥5.0 cm for women, must be accurately assessed to ensure appropriate patient selection for repair [5].

Recognizing these challenges, international guidelines recommend standardizing AAA measurement techniques to reduce variability and improve clinical consistency. A uniform protocol for caliper placement, combined with structured reporting guidelines, may enhance reproducibility across imaging centers and practitioners [41]. Furthermore, guidelines advocate for integrating AI and automated imaging analysis to minimize human error. AI-assisted measurement tools can provide objective, consistent, and reproducible aneurysm size assessments, reducing interobserver variability and improving patient outcomes [6].

Evolution in screening strategies

Impact of Revised Screening Recommendations

The SVS and ESVS have introduced significant revisions to AAA screening recommendations, reflecting changes in epidemiology and advancements in diagnostic accuracy. Previous guidelines strongly recommended universal screening for men aged 65 and older. However, recent epidemiological data indicate a declining prevalence of AAA, largely due to reduced smoking rates and improved cardiovascular risk management​ [6,16].

Given these trends, the contemporary recommendations shift towards a more selective screening approach, prioritizing high-risk individuals. This includes men aged 65+ who have ever smoked, first-degree relatives of AAA patients, and individuals with peripheral aneurysms. While general screening for men remains recommended, the definition of high-risk groups is now tailored to country-specific conditions, including AAA prevalence and healthcare infrastructure​ [6,16].

Given the significantly lower prevalence, the guidelines also acknowledge the limited evidence for AAA screening in women. Studies indicate that AAA prevalence in women is nearly fourfold lower than in men, and the mortality risk associated with surgical repair is higher. As a result, routine population screening for women is not recommended. Instead, screening is suggested only for women with a first-degree relative who has AAA, as familial clustering is associated with a higher risk​ [6,16].

Additionally, international guidelines emphasize incidental detection as an important factor in modern screening strategies. Advances in imaging for other conditions (e.g., CT for back pain or abdominal discomfort) have led to increased incidental diagnoses of AAA. The guidelines now strongly recommend referral to a vascular surgeon for any incidentally detected AAA, except in cases of very limited life expectancy​ [6,16].

Cost-Effectiveness of Population-Based vs. Targeted Screening

One of the key considerations in screening recommendations is the cost-effectiveness of screening programs. Population-based screening remains highly cost-effective in certain countries, particularly where the prevalence of AAA in men aged 65+ remains above 1% [19]. However, a more selective screening approach targeting high-risk individuals is considered more efficient in countries where prevalence has fallen below this threshold.

Data from long-term screening trials in the UK and Sweden indicate that screening continues to reduce AAA-related mortality [19,42]. However, the absolute number of aneurysms detected has declined, raising concerns about diminishing returns in low-prevalence settings. Screening programs should be periodically re-evaluated to ensure cost-effectiveness based on current epidemiological trends.

Targeted screening is increasingly favored as a cost-effective alternative to universal screening. Studies indicate that focusing on smokers, individuals with a family history of AAA, and those with existing vascular disease yields a higher detection rate per screened individual, making it a more economically viable strategy. Additionally, screening during routine cardiovascular assessments for patients with coronary artery disease or carotid stenosis has been proposed as a feasible approach to identify undiagnosed cases​ [22].

Integration of Genetic and Biomarker-Based Risk Stratification

The growing role of genetic and biomarker-based risk stratification in AAA screening cannot be overestimated. While traditional screening criteria rely on factors such as age, sex, and smoking history, recent research suggests that genetic predisposition plays a significant role in AAA development [43]. Studies indicate that first-degree relatives of AAA patients have a twofold higher risk, and genetic mutations affecting connective tissue integrity are associated with increased susceptibility​ [44].

Emerging technologies now allow for comprehensive genetic testing, which can help identify high-risk individuals even before aneurysm formation. Conditions such as Marfan syndrome, Loeys-Dietz syndrome, and vascular Ehlers-Danlos syndrome have well-documented associations with aortic pathology, and targeted screening in these populations is increasingly recommended​ [45].

Beyond genetics, biomarker research is advancing rapidly, with several promising circulating markers being investigated for AAA risk assessment. Biomarkers linked to inflammation, extracellular matrix degradation, and vascular remodeling, such as C-reactive protein, interleukin-6, matrix metalloproteinases, osteopontin, fibrillin-1, and elastin fragments, may improve the ability to predict AAA growth and rupture risk, allowing for more personalized screening protocols [46].

Given these developments, international guidelines recommend continued research into integrating genetic screening and biomarker analysis into AAA risk stratification models [33]. Although these approaches are not yet widely implemented in routine practice, they represent a potential paradigm shift in AAA screening, moving toward personalized medicine and precision diagnostics.

Clinical decision-making: a shift in practice

How Improved Imaging Techniques Influence Treatment Thresholds

Advances in imaging technology are reshaping treatment thresholds for AAA management. Historically, the decision to proceed with elective repair was based on aneurysm diameter alone, typically ≥55 mm for men and ≥50 mm for women. However, improved imaging modalities such as high-resolution CTA, MRA, and AI-assisted risk prediction models now allow for a more nuanced rupture risk assessment​ [10-13].

Newer imaging techniques can assess aortic wall integrity, thrombus burden, and biomechanical stress, which provide additional predictive value beyond diameter alone. Studies have demonstrated that aneurysms with heterogeneous thrombus composition, increased wall stress, or rapid expansion (>10 mm per year) have a higher rupture risk, even if they are below the traditional surgical threshold [47]. Consequently, recent guidelines support a more individualized approach to deciding when to intervene, incorporating advanced imaging parameters alongside traditional size criteria​ [6,16].

Furthermore, for patients with genetically linked AAAs, such as those with Loeys-Dietz syndrome or vascular Ehlers-Danlos syndrome, the guidelines recommend earlier intervention at smaller diameters due to the higher rupture risk associated with these conditions​ [45]. This personalized approach ensures that high-risk patients are treated earlier while low-risk patients are spared unnecessary surgery.

Role of Imaging in Shared Decision-Making With Patients

Shared decision-making (SDM) has become an essential component of AAA management, recognizing that deciding to proceed with surgery is often complex and depends on patient preferences, life expectancy, and comorbidities [45]. Contemporary guidelines highlight SDM as a core principle, emphasizing the need for comprehensive patient education, decision aids, and multidisciplinary discussions.

SDM is particularly relevant for patients with small AAAs who are candidates for surveillance rather than immediate surgery. Patients should be informed of the natural history of AAA, potential risks of rupture, and associated risks with elective repair [48]. The guidelines recommend using decision support tools, including visual aids, interactive risk calculators, and AI-generated rupture risk estimates, to help patients make informed choices about their treatment​ [6].

A recent study found that patients who are actively involved in the decision-making process experience reduced decisional conflict, greater satisfaction with care, and improved adherence to surveillance protocols [48]. International guidelines recommend integrating structured SDM frameworks into AAA management, ensuring that patients receive clear, unbiased information before committing to treatment​ [6].

Integrating Multidisciplinary Assessment for Complex Cases

The management of complex AAA cases, including inflammatory aneurysms, ruptured AAAs, or patients with significant comorbidities, requires a multidisciplinary team (MDT) approach. All complex AAA cases should be evaluated by an MDT comprising vascular surgeons, interventional radiologists, anesthetists, cardiologists, and geneticists, particularly for patients with underlying genetic disorders or high surgical risk​ [49].

The guidelines emphasize that MDT discussions should include detailed imaging reviews, assessing factors such as aneurysm morphology, endovascular repair feasibility, and individualized rupture risk assessments. In cases where open surgery and endovascular repair are both options, an MDT approach ensures that the best treatment modality is chosen based on patient-specific factors​ [6,16].

Additionally, the MDT should evaluate nonoperative management options for patients with borderline surgical indications, such as optimized medical therapy and lifestyle modifications. For all AAAs that require immediate intervention, an MDT discussion can help balance the risks and benefits of surgical vs. conservative approaches [49].

## Conclusions

Advancements in imaging have revolutionized the diagnosis and management of AAA. While US remains the primary screening tool, advanced modalities like CTA, MRA, and AI-assisted imaging enhance risk stratification and early detection. Population-based screening trials in the UK, Denmark, and Australia confirm a significant reduction in AAA-related mortality, supporting structured screening programs. However, shifting epidemiological trends necessitate a more targeted screening approach, prioritizing high-risk individuals.

Standardizing imaging protocols is crucial to ensuring accurate aneurysm measurement and optimal intervention timing. Emerging technologies, including AI, IVUS, and PET-CT, provide new insights into rupture risk assessment. Future research should explore genetic markers and biomarkers for personalized screening. As AAA prevalence declines, updated guidelines must align with technological innovations to optimize patient outcomes while maintaining cost-effectiveness. A multidisciplinary, precision-based approach will continue to shape the evolving landscape of AAA management.
